# Characterization of polymer-polymer type charge-transfer (CT) blend membranes for fuel cell application

**DOI:** 10.1016/j.dib.2018.02.031

**Published:** 2018-02-15

**Authors:** Shiyan Feng, Shoichi Kondo, Takahiro Kaseyama, Taichi Nakazawa, Takamasa Kikuchi, Roman Selyanchyn, Shigenori Fujikawa, Liana Christiani, Kazunari Sasaki, Masamichi Nishihara

**Affiliations:** a Graduate School of Engineering, Kyushu University, Fukuoka 819-0395, Japan; b Nissan Chemical Industries, Ltd., Japan; c World Premier International Research Center Initiative, International Institute for Carbon-Neutral Energy Research (WPI-I2CNER), Kyushu University, Fukuoka 819-0395, Japan; d Center of Innovation, Center for Co-Evolutional Social Systems (COI-CESS), Kyushu University, Fukuoka 819-0395, Japan; e Next-generation Fuel Cell Research Center (NEXT-FC), Kyushu University, Fukuoka 819-0395, Japan

## Abstract

The data presented in this article are related to polymer-polymer type charge-transfer blend membranes for fuel cell application. The visible spectra of the charge-transfer (CT) blend membranes indicated formation of CT complex in the blend membranes, and behavior of CT complex formation by polymers was clarified by Job plot of the visible spectra. The effect of fluorine for membrane property and fuel cell performance of CT blend membranes were evaluated by ^19^F NMR and overvoltage analysis, respectively.

**Specifications Table**

**Table:** 

Subject area	*Materials Sciences*
More specific subject area	*Polymer electrolyte membrane*
Type of data	*Figures and scheme*
How data was acquired	*Visible spectroscopy,*^*19*^*F NMR, proton conductivity, impedance measurement with fuel cell test*
Data format	*Raw data*
Experimental factors	*Heat treatment was carried out for some samples.*
Experimental features	*The thin CT membrane had a similar resistance comparing with Nafion 212 membrane.*
Data source location	*Next-generation Fuel Cell Research Center (NEXT-FC), Kyushu University, Fukuoka, Japan*
Data accessibility	*Data is with the article.*

**Value of the data**

• Visible spectra indicated that charge-transfer (CT) complex was formed between distinct polymers in membranes.
• Integration of all fluorine peaks in ^19^F NMR were roughly same between before and after heat treatment.
• Overvoltage evaluation of CT blend membranes indicated that the thin CT membrane had a similar resistance comparing with Nafion 212 membrane.

## 1 Data

The dataset of this article provides CT formation in the blend membranes, effect of heat treatment to fluorine and overvoltage evaluation from fuel cell test using CT blend membranes.

## 2 Experimental design, materials, and methods

### 2.1 Synthesis of non-fluorinated sulfonated polyimide (nf-SPI) and aromatic polyether (A-PE)

**Scheme 1:**
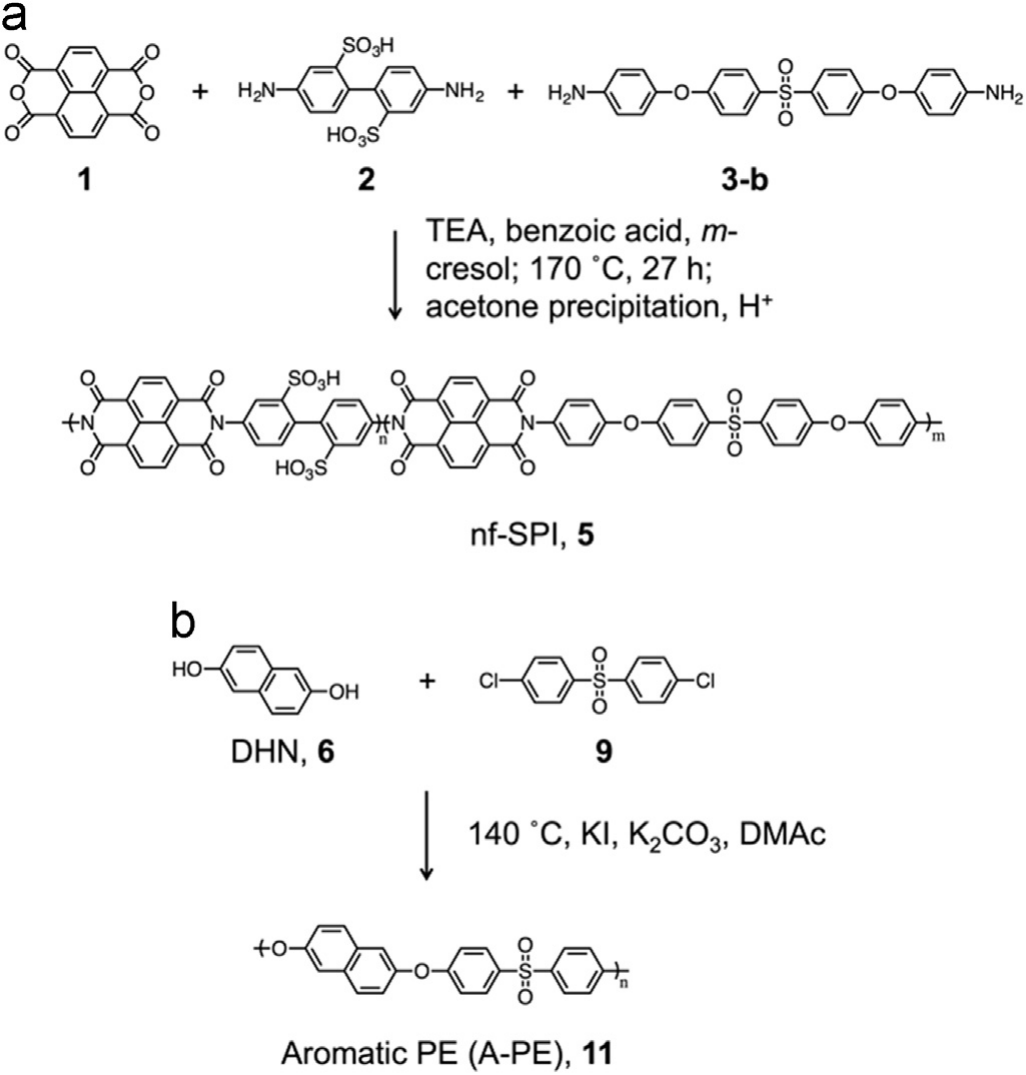
Synthesis of (a) non-fluorinated sulfonated polyimide (nf-SPI) containing electron-deficient NDI, (b) aromatic polyether containing electron-rich DHN.

Nf-SPI was synthesized by the same process and reported also in other literature [1]. The synthesis of A-PE was carried out as reported in the literature [2]. ( Scheme 1).

### 2.2 Visible transmittance spectroscopy of CT blend membranes

**Fig. 1:**
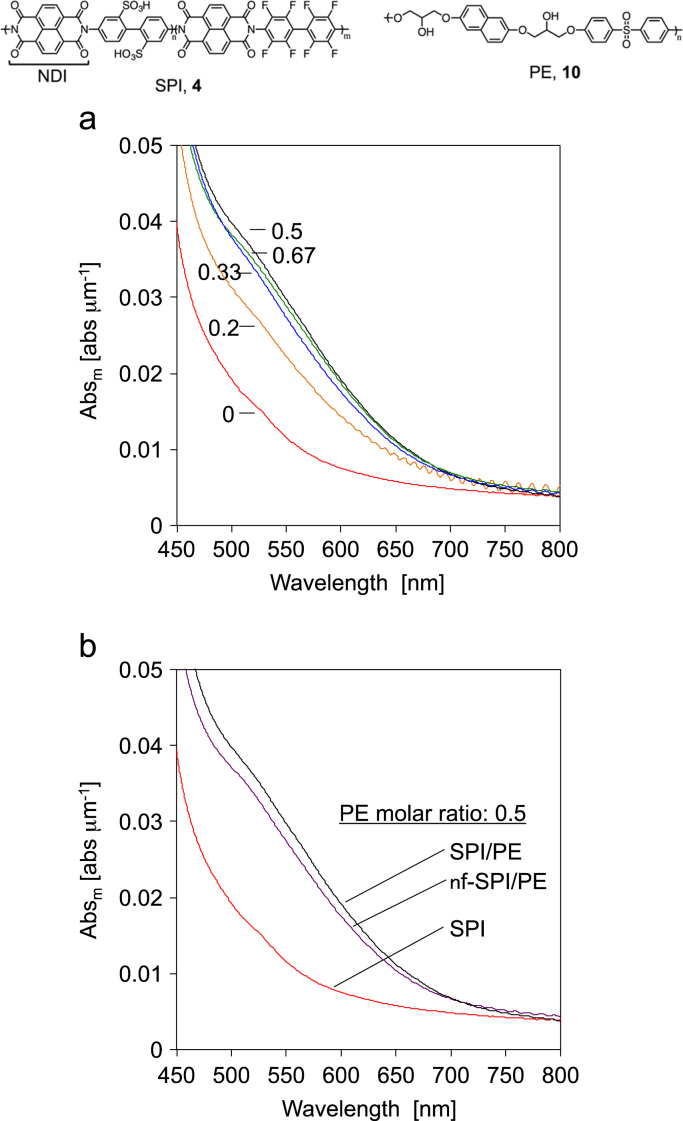
(a) Normalized visible spectra of SPI **4** and SPI/PE **10** membranes. (b) Visible spectra of SPI and nf-SPI/PE 0.5 membranes.

**Fig. 2:**
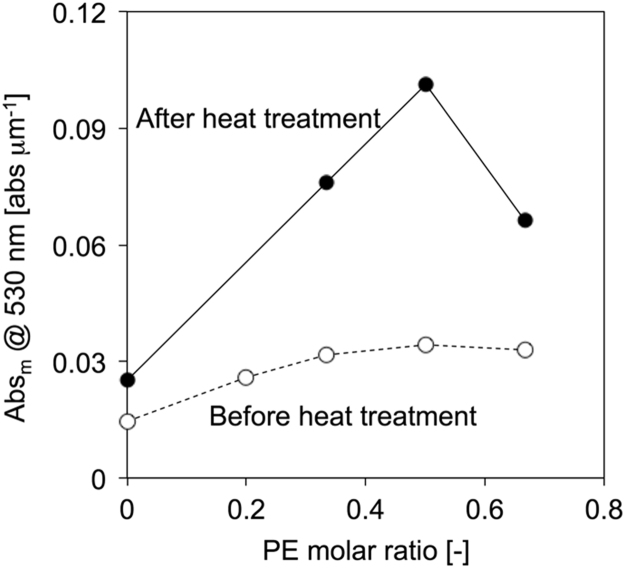
Job plot of SPI/PE membranes before (open circle) [3] and after (solid circle) heat treatment calculated from normalized absorbance at 530 nm.

Visible spectra of the blend membranes were measured with a V-650 instrument, an ISV-722 integrating sphere and a SSH-506 sample holder (JASCO, Japan). The Abs as film absorbance was calculated as below (Fig. 1, Fig. 2):

$$\mathrm{Abs}={\mathrm{Abs}}_{m}/l$$

where Abs is the absorbance normalized by thicknesses of membranes, Abs_m_ is the measured absorbance, *l* is the thickness of membrane.

Job plot of CT blend membranes ware prepared from PE molar ratio in the CT blend membranes and the normalized absorbance at 530 nm, which was assigned as CT absorption.

### 2.3 ^19^F NMR of CT blend membrane in DMSO-d_6_

**Fig. 3:**
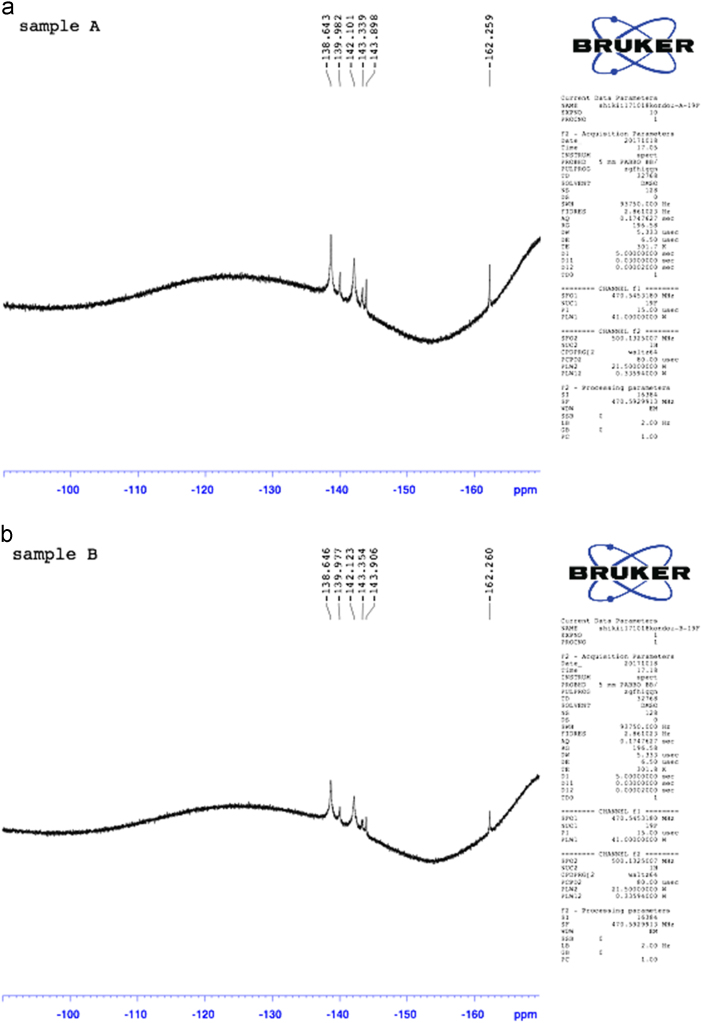
The ^19^F NMR of SPI/PE 0.5 membrane (a) before heat treatment, (b) after heat treatment at 130 °C for 2 h (DMSO-d_6_).

^19^F NMR of SPI/PE CT blend membranes was measured using the samples before and after heat treatment. Heat treatment was carried out at 130 °C for 2 h in vacuo (Fig. 3).

### 2.4 Weight loss (WL) of CT blend membrane

**Fig. 4:**
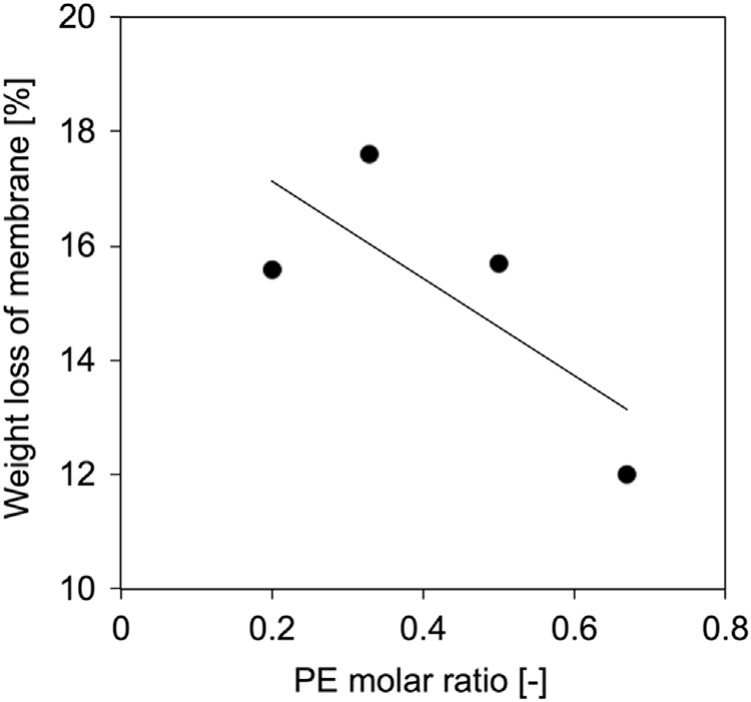
Weight loss of SPI/PE membranes as a function of PE molar ratio.

Weight loss (WL) of membrane was calculated as below;

$$\mathrm{WL}\;\mathrm{of}\;\mathrm{membrane}=\left(W_{d-\text{before}}–W_{d-\text{after}}\right)/W_{d-\text{before}}\times 100,$$

where *W*_d-before_ and *W*_d-after_ are the dry masses of the samples before and after experiment, respectively (Fig. 4).

### 2.5 Proton conductivity of Nafion 212

**Fig. 5:**
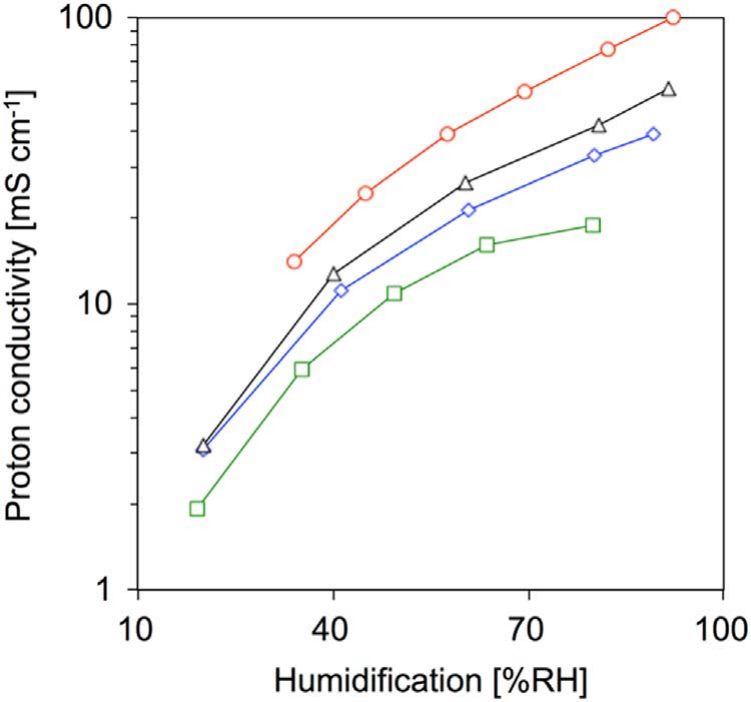
Proton conductivity of Nafion 212 at different temperature and humidification. □: 30 °C; ◇: 60 °C; △: 80 °C; ◯: 120 °C.

Through-plane proton conductivities of all membranes were measured by a membrane test system (MTS-740, Scribner) under different temperatures and humidities [3]. The proton conductivity (σ) was calculated as below (Fig. 5);

$$\sigma =l/\left(R_{m}\times A\right),$$

where *l* is the thickness of membranes between two Pt electrodes, *A* is overlap area of membranes between the two electrodes, and *R*_m_ is the resistance of the membranes obtained from the high frequency intercept of the real axis in the Nyquist plots of the impedance spectra.

### 2.6 Overvoltage evaluation of CT blend membrane

**Fig. 6:**
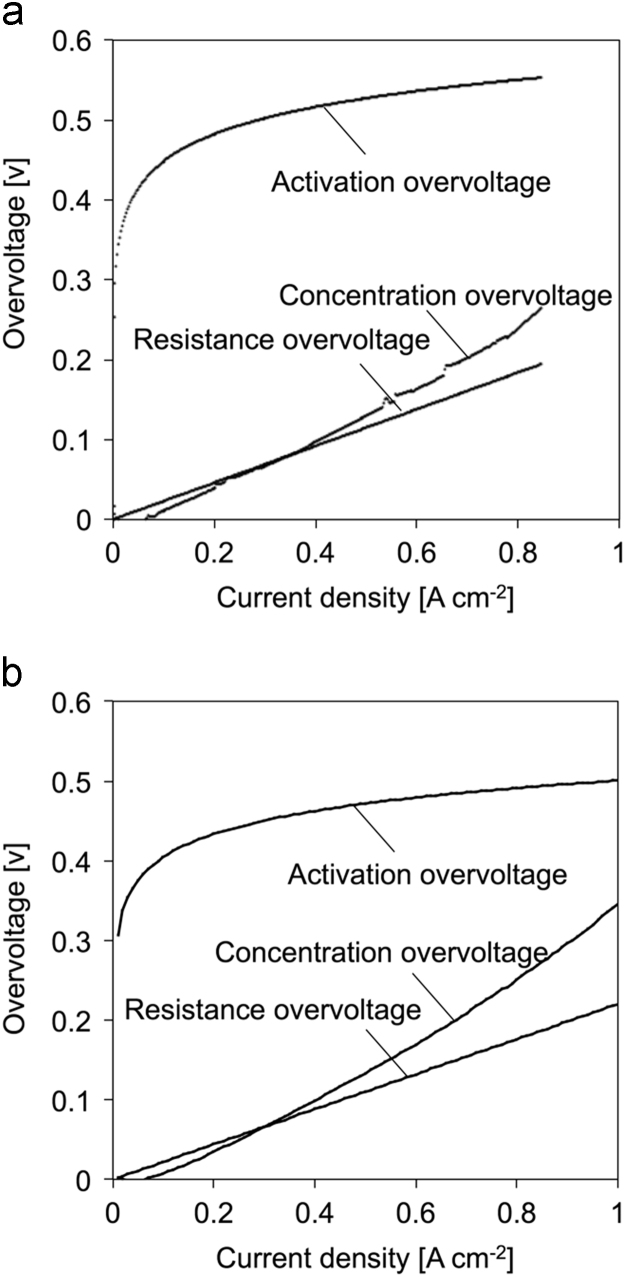
Overvoltage of SPI/PE 0.33 membrane (a) and Nafion 212 (b).

A membrane-electrode assembly (MEA) was fabricated from the CT blend membrane and Nafion 212, and a single cell test was performed following the Ref. [3]. The overvoltage was calculated based on polarization curve and cell impedance according to NEDO (New Energy and Industrial Technology Development Organization, Japan) protocol [4] (Fig. 6).
